# Incidence and causes of traumatic and non-traumatic spinal cord injury in Salzburg, Austria: a multi-center hospital network–based study

**DOI:** 10.3389/fneur.2026.1728274

**Published:** 2026-02-06

**Authors:** Mahdi Safdarian, Aljoscha Thomschewski, Stefan Leis, Laura Schnetzer, Georg Zimmermann, Wolfgang Voelckel, Thomas Freude, Eugen Trinka

**Affiliations:** 1 Department of Neurology, Neurocritical Care and Neurorehabilitation, Christian Doppler University Hospital, Centre of Cognitive Neuroscience, Member of EpiCARE, Paracelsus Medical University, Salzburg, Austria; 2 Neuroscience Institute, Christian Doppler University Hospital, Centre of Cognitive Neuroscience, Paracelsus Medical University, Salzburg, Austria; 3 Vorarlberg Institute for Vascular Investigation and Treatment, Feldkirch, Austria; 4 Team Biostatistics and Big Medical Data, IDA Lab Salzburg, Paracelsus Medical University, Salzburg, Austria; 5 Research Program Biomedical Data Science, Paracelsus Medical University, Salzburg, Austria; 6 Department of Artificial Intelligence and Human Interfaces, Paris Lodron University, Salzburg, Austria; 7 Department of Anesthesiology and Intensive Care Medicine, AUVA Trauma Center Salzburg, Salzburg, Austria; 8 University Hospital for Orthopaedics and Traumatology, Paracelsus Medical University, Salzburg, Austria; 9 Karl Landsteiner Institute for Clinical Neuroscience, Salzburg, Austria

**Keywords:** causes, epidemiology, incidence, Salzburg, spinal cord injuries

## Abstract

**Introduction:**

Spinal cord injury (SCI) causes substantial disability and healthcare burden. While Austrian data are available for traumatic SCI, incidence and causes of non-traumatic SCI have not been reported previously. This study aims to determine the incidence and causes of traumatic and non-traumatic SCI in Salzburg, Austria, from 2013 to 2023.

**Methods:**

We retrospectively screened hospital databases from Salzburg County Hospitals (SALK) and the regional trauma hospital (AUVA) using an ICD-based algorithm. This hospital network captures the majority of SCI care in the region; however, cases managed exclusively outside this network may not be captured. Cases were included if SCI was confirmed by MRI or medical documentation and the index event occurred during 2013–2023. Data were extracted into a validated REDCap instrument. Incidence was calculated for Salzburg residents; non-resident cases were analyzed separately for healthcare burden.

**Results:**

A total of 587 SCI cases were identified, including 99 traumatic (16.8%) and 488 non-traumatic (83.2%). The average incidence was 9.7/100,000/year (traumatic: 1.6; non-traumatic: 8.1). The cohort had a median age of 62 years; male-to-female ratio was 2.96 for traumatic and 1.02 for non-traumatic cases. Falls (52.5%) were the leading traumatic cause, followed by transport (24.2%) and sports injuries (19.2%). Multiple sclerosis (24.8%) was the most frequent non-traumatic etiology, followed by degenerative disorders, neoplasms, and infections. Nearly half of all lesions were cervical, with C5–C8 most commonly affected. From 2020 onward, SCI incidence declined sharply, temporally coinciding with the COVID-19 pandemic.

**Conclusion:**

SCI incidence in Salzburg (9.7/100,000) was higher than earlier Austrian estimates due to inclusion of non-traumatic cases, which comprised over 80% of all SCIs. Falls and degenerative/inflammatory conditions were major contributors. Preventive strategies should address both traumatic and non-traumatic causes, and a prospective national registry is warranted.

## Introduction

Spinal cord injury (SCI) is a major global health concern, leading to substantial disability and socioeconomic burden (1). A recent study of the Global Burden of Disease (GBD) project revealed that approximately 20.6 million individuals were living with SCI worldwide, with 0.9 million new cases reported each year (2). The Federal State of Salzburg represents a well-defined geographic and healthcare region with centralized tertiary care structures for neurological and trauma services. The majority of patients with suspected spinal cord injury in Salzburg are managed within a small number of referral hospitals, making the region particularly suitable for hospital network–based epidemiological analyses. Salzburg County has a population of approximately 560,000 inhabitants (3).

Since curative treatments for most SCIs are lacking, understanding their epidemiology and etiology is essential for prevention (4). Traumatic SCIs usually result from motor vehicle accidents, falls, or sports injuries, while non-traumatic SCIs stem from conditions such as degenerative spine disease, infections, autoimmune disorders, multiple sclerosis, or tumors (5). In Austria, a study analyzing data from 2002 to 2012 reported an annual average age-standardized incidence rate of 13.98 per million population for traumatic SCI (6). This study found a higher risk among individuals aged over 65 years, with falls being the predominant cause of traumatic SCI in this age group (6). However, there is still a huge gap in the epidemiological, especially region-specific data on both traumatic and non-traumatic SCI from Austria. Comparable European studies provide context for regional incidence patterns in high-income settings. In Finland, the mean annual incidence of traumatic SCI was reported as approximately 36.6 cases per million inhabitants per year, with falls and transport accidents as leading causes, highlighting the importance of age-related injury mechanisms in northern European populations (7). A large registry-based analysis from Germany estimated an overall SCI incidence of 15.7 per million population per year, consistent with Western European trends for traumatic injury (8). Such regional estimates emphasize that SCI incidence varies across high-income European countries and underscore the need for local data to inform prevention and healthcare planning. Epidemiological studies are critical for distinguishing between these causes, especially in regions where non-traumatic SCIs may be underdiagnosed or misclassified (9). This study bridges this gap by providing an analysis of the incidence and causes of SCIs in the Federal State of Salzburg, Austria, thereby informing future healthcare strategies. To this end, we systematically compiled a database of all patients diagnosed with SCI from 2013 to 2023, collecting incidence data across the major regional hospital network and categorizing cases by cause. Beyond incidence estimation, characterization of acute clinical variables such as ICU utilization and associated injuries provides context regarding the immediate healthcare burden of SCI, which is particularly relevant for regional health system planning.

## Methods

### Study design, setting, and ethics

This retrospective multicenter epidemiological study is part of the EPIDEMSCI project (“Epidemiological evaluation of incidence, mortality, and life expectancy in traumatic and non-traumatic spinal cord injuries in the Federal State of Salzburg, Austria: 2013–2023”), conducted under study protocol number V6, 2021-04-20. The project was approved by the Ethics Committee for the State of Salzburg (EK Nr: 1023/2021). All applicable institutional and governmental regulations regarding the ethical use of patient-identifiable data were strictly followed, and full compliance with the DSGVO (European Data Security Act) was ensured at all participating centers. The study represents a retrospective epidemiological analysis of existing patient data; no clinical trial or interventional component was included. Data acquisition was performed exclusively in digital form, limited to patient files. Methodology and results were reported in line with the recommendations of the Strengthening the Reporting of Observational Studies in Epidemiology (STROBE) guidelines (10).

### Data sources and case ascertainment

To ensure comprehensive case ascertainment of SCI from the hospital databases, first we developed an ICD code list based on the current literature, expert panel consensus, and our own previous clinical works (11–13). This list conveys more than 200 ICD-9 and -10 Codes of all etiologies, which could possibly contain cases of SCI (Supplementary material). We then used the ICD code list from the GBD project data team, in addition to comments from our colleagues in the National Registry of Spinal Cord Injury in Iran (NSCIR-IR) to modify and improve our list.

A specific REDCap data set was developed for this project by our team based on a comprehensive review of existing SCI registries, incorporating both traumatic and non-traumatic domains (14). The development process has been described in detail elsewhere (14). In summary, for traumatic SCI, we used the WHO International SCI Data Sets as the primary foundation, supplemented and refined with elements from the Australian Spinal Cord Injury Registry (ASCIR), the Rick Hansen Spinal Cord Injury Registry (RHSCIR), the European Multicenter Study on Spinal Cord Injury (EMSCI), and the National Spinal Cord Injury Registry of Iran (NSCIR-IR). For non-traumatic SCI data, we followed classification from International Spinal Cord Society (ISCoS) (15). These were merged into a structured REDCap instrument comprising 79 variables, which is available for online use in the REDCap Shared Library under the name *‘Epidemiologic studies of Spinal Cord Injury: Traumatic and Non-Traumatic’* and in Supplementary material.

### Study population and data collection

**Figure 1:**
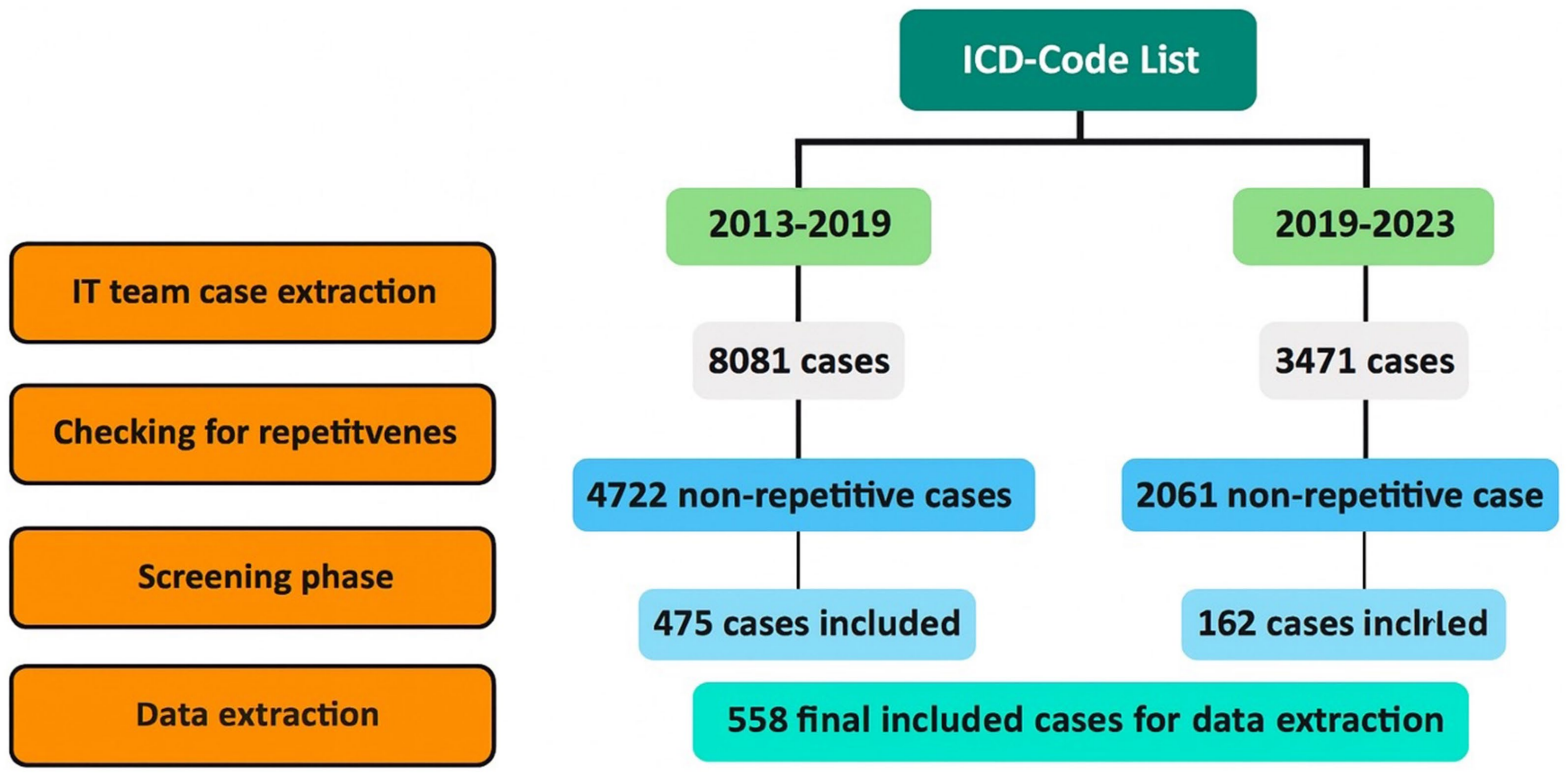
Screening flowchart for the SALK cases.

This study was conducted at the Department of Neurology, Christian-Doppler University Hospital Salzburg, Austria, and aimed to retrospectively collect epidemiological data on traumatic and non-traumatic SCI in the northern catchment area of Salzburg (VR 51: Salzburg-Stadt, Flachgau, Tennengau). This comprises the majority of the county’s population and includes both urban and rural settings. As most tertiary care and referral structures for Salzburg are located in this region, it provides a representative sample for the entire county. Data were obtained from the medical databases of the Salzburg County Hospitals (LKH and CDK, mainly non-traumatic cases) and from Unfallkrankenhaus Salzburg (AUVA, the regional referral trauma hospital) for traumatic cases. Using our ICD code list, the SALK IT team retrieved cases from 2013 to 2019 and 2019–2023 (Figure 1). Lesion level distribution was reported at the lesion level to reflect multilevel spinal cord involvement; however, incidence estimates, and all patient counts are reported at the patient level. Figure 1 summarizes the stepwise ICD-based case identification, screening, and inclusion process.

### Case definitions

**Figure 2:**
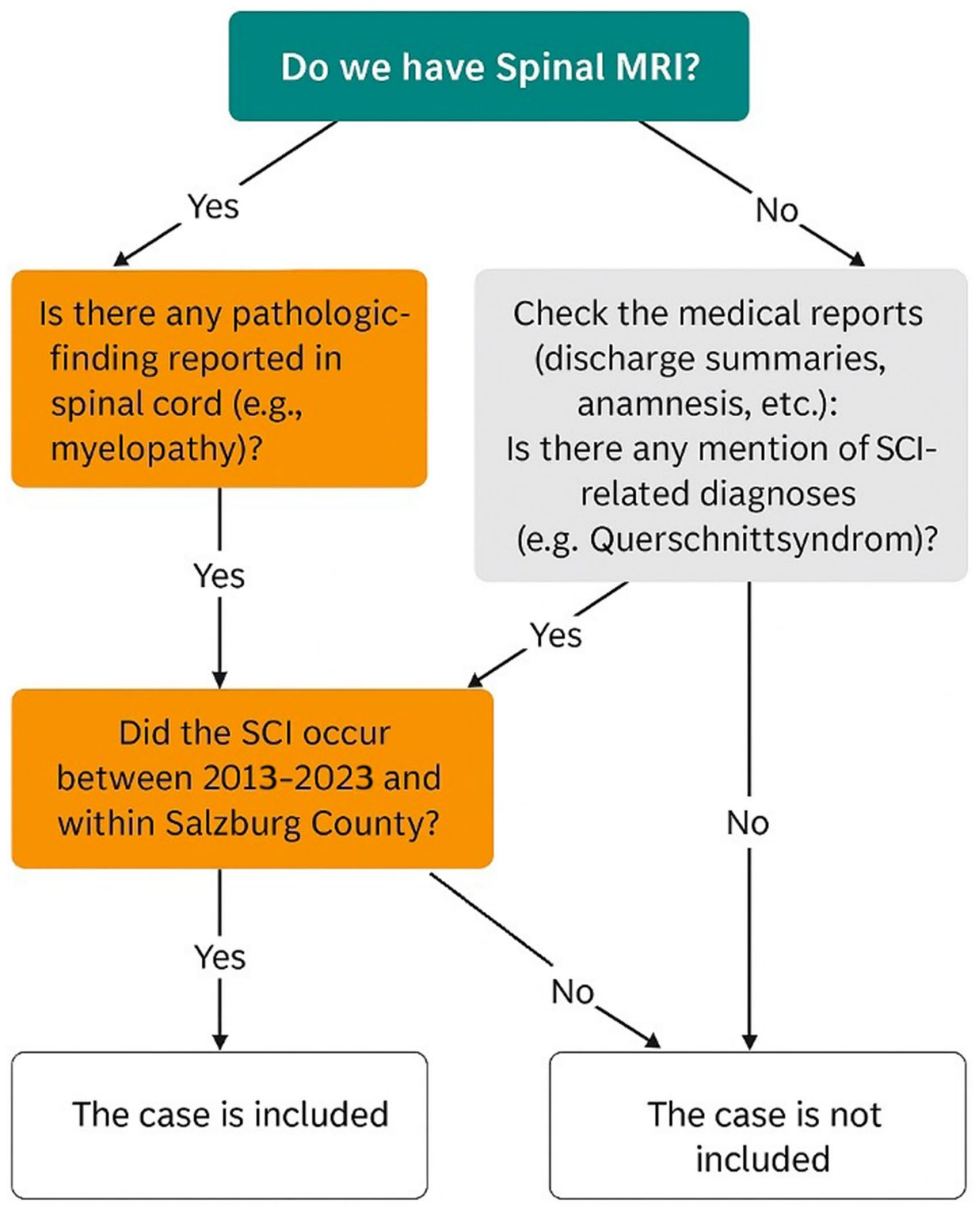
Screening algorithm for non-traumatic SCI cases.

After removing duplicates, two researchers independently screened all cases using our internally developed algorithm. All cases identified through ICD-based screening underwent manual verification by the two investigators using a predefined classification algorithm, with discrepancies resolved by consensus. According to this screening algorithm, for Salzburg citizens (according to the living address) if spinal MRI was available, cases were included when pathological findings (e.g., myelopathy) were present, and the index event occurred during 2013–2023. If no MRI was available, medical reports (e.g., discharge summaries, anamnesis) were reviewed for SCI-related diagnoses, which were then assessed using the same time and location criteria. Non-traumatic spinal cord injury was defined according to the International Spinal Cord Injury Data Sets for Non-Traumatic SCI (New & Marshall). Multiple sclerosis cases were included only when spinal cord involvement was documented on MRI and considered clinically relevant in the treating physician’s assessment; isolated incidental MRI findings without clinical relevance were not classified as NTSCI. Cases not meeting these conditions were excluded (Figure 2). Traumatic cases were identified from the Trauma Registry of the German Society for Trauma Surgery (TR-DGU). All the traumatic SCI cases treated in AUVA had been registered in the TR-DGU. For traumatic SCI cases, we included cases who were Salzburg citizens (according to living address at the time of injury). We also collected data of traumatic cases registered in AUVA but were not Salzburg citizens, which were not included in this study. Non-resident traumatic SCI cases treated within the regional hospital network were analyzed separately to reflect healthcare burden. For those Salzburg citizens whose traumatic injury may occurred outside the Salzburg county, we collected their data according to the search in CDK and LKH hospitals rehabilitation centers in neurology and Orthopedic departments. For non-traumatic spinal cord injury, we considered the first documented hospital presentation with clinical or MRI evidence of spinal cord involvement during the study period as the index event. A formal washout period prior to 2013 was not feasible due to incomplete pre-2013 data across all sources. All data were drawn from hospital databases (ORBIS, Agfa®) and transferred into REDCap instrument. A sample dataset is provided in Supplementary material.

### Residence-based incidence definition

Incidence estimates were calculated based on the place of residence at the time of diagnosis or injury, rather than the geographic location of injury or treatment. This approach is particularly appropriate for non-traumatic SCI, which typically develops over time and is not linked to a discrete injury location. Salzburg residents sustaining traumatic SCI outside the county and managed entirely outside the regional hospital network may not have been captured.

### Statistical methods

Basic clinical and demographic characteristics were summarized using mean and standard deviation (SD), or median and interquartile range (IQR), for metric variables, as appropriate, and counts and percentages for categorical variables, respectively. Incidences are reported with corresponding two-sided 95% Agresti-Coull confidence intervals (CI) for proportions. Since the primary aim of the study was to estimate incidences and distributions of certain variables, no statistical hypothesis tests were conducted.

## Results

### Collected data

We identified 11,552 cases between 2013 and 2019 (8,081 IDs) and 2019–2023 (3,471 IDs), corresponding to 6,783 unique patients from LKH and CDK University Hospitals. After removal of repetitive encounters, 4,722 non-repetitive SALK cases remained, of which 475 met screening criteria and 558 were retained for full data extraction (Figure 1). The main reasons for exclusion during screening were absence of confirmed SCI (rule-out diagnoses), duplicates or re-admissions, residence outside Salzburg County (for incidence calculations), and cases outside the 2013–2023 inclusion period. An additional 195 traumatic cases were retrieved from the AUVA and TR-DGU databases, of which 102 were eligible. Altogether, after two rounds of screening, a total of 587 SCI cases were included in the final cohort.

### Demographic data

**Figure 3:**
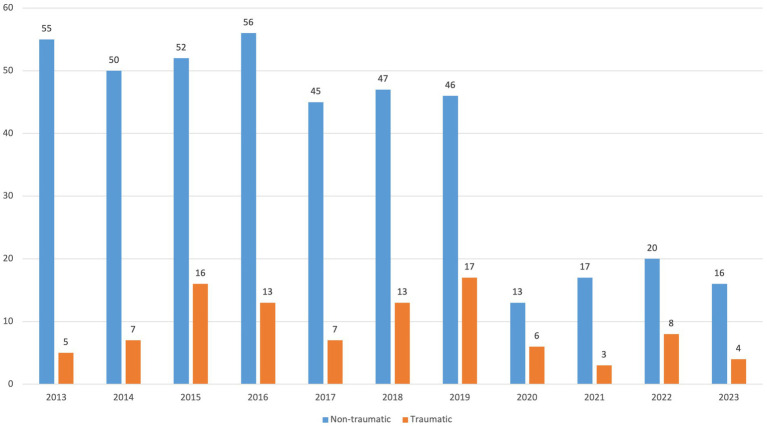
Distribution of the cases according to the type of injury during the years 2013–2023.

Of the 587 cases, 99 (16.8%) were traumatic and 488 (83.2%) non-traumatic (Figure 3). On average, 47 cases were recorded annually (9 traumatic, 38 non-traumatic). The cohort included 321 men (54.7%) and 266 women (45.3%), with a median age of 62 years (IQR 48–73). The male-to-female ratio was 2.96 for traumatic and 1.02 for non-traumatic cases. Most patients were Austrian (92%), while 8% were of other nationalities. Geographically, 25% of traumatic and 38% of non-traumatic cases occurred in Salzburg city, the rest in surrounding districts. Regarding marital status, 45.3% were married, 22.3% single, 10.0% divorced, 9.1% widowed, and 11.9% unknown.

### Incidence data

From 2013 to 2023, the average incidence of SCI among Salzburg residents was 9.7 per 100,000 (95% CI 7.4–12.7). Stratified by cause, incidence was 1.6 (95% CI 0.8–3.2) for traumatic and 8.1 (95% CI 6.0–10.8) for non-traumatic SCI (Table 1).

**Table 1:** Average incidence of SCI per age group (per 100.000).

Age group	Incidence	95% CI
0–14 y	0.3	0.0–6.3
15–29 y	4.2	1.3–11.1
30–44 y	4.7	1.7–11.2
45–59 y	9.4	5.1–16.7
60–74 y	17.5	10.4–29.0
75–89 y	22.0	11.1–41.8
90+ y	6.0	0.0–111.8

### Injury related data

#### Traumatic injuries

Among 99 traumatic SCIs, the main causes were falls (52.5%), transport accidents (24.2%), and sports injuries (19.2%). Single cases were due to gunshot and occupational injury, while no incidents were related to drowning, fire, assault, suicide, or disasters. Within transport injuries, car crashes (*n* = 8), cycling (*n* = 9), and motorcycling (*n* = 4) were most frequent, with no pedestrian or heavy-vehicle accidents. Sports-related injuries included skiing (*n* = 7), mountain/downhill biking (*n* = 4), hiking (*n* = 3), paragliding (*n* = 2), rock climbing (*n* = 1), and two other activities. Injury locations were most often homes (*n* = 23), streets/highways (*n* = 22), and sports facilities (*n* = 12). The leading activities at time of injury were sports (*n* = 16), leisure (*n* = 20), and work (*n* = 12). Most injuries were blunt (92 cases), with only four penetrating. Temporal patterns showed 38.8% of injuries occurred on weekends and 61.2% on weekdays. September had the highest incidence (12.9%), with additional peaks in June and August, while February had the fewest cases. Prehospital data recorded 16 cardiac arrests with CPR and 26 intubations. Immobilization was applied in 52 cervical and 9 spinal cases. Associated injuries included 47 traumatic brain injuries, 20 extremity fractures, and 14 internal organ injuries.

#### Non-traumatic injuries

Of the 488 non-traumatic SCIs in our study, 484 (99.2%) were acquired abnormalities and 4 (0.8%) congenital or genetic disorders (Table 2). Acquired causes were dominated by inflammatory and autoimmune diseases (30.8%), degenerative spine disorders (24.4%), neoplasms (17.4%), and infections (17.2%). Multiple sclerosis was the single most frequent etiology (121 cases, 24.8%). Spinal stenosis and disc prolapse were the leading degenerative causes. Within the neoplastic group, 58.3% were benign (mainly intradural/extramedullary tumors such as meningioma, schwannoma, and neurofibroma), while malignant cases were mostly vertebral metastases (51.4%). Vascular causes were primarily ischemic (71.1%). Among infections, bacterial agents were predominant (81.9%), with *Staphylococcus aureus* responsible for more than half of extradural abscesses. Rare etiologies included a single metabolic case (vitamin deficiency) and no toxic, radiation-related, or parasitic causes. Congenital cases were limited to four patients (two spinal dysraphisms and two Arnold–Chiari malformations). Symptom onset (*n* = 416) was acute (less than 1 day) in 12.0%, sub-acute (1–7 days) in 14.9%, prolonged (7 days to 1 month) in 24.8%, and gradual (more than 1 month) in 48.3%, reflecting the typically chronic course of many non-traumatic SCIs.

**Table 2:** Classification and distribution of the non-traumatic cases in our study (number, %).

Aquired abnormalitites (483, 99.2%)	Vertebral column degenerative disorders (118, 24.4%)^#^	Spinal stenosis (61, 51.7%)	
		Disc prolapse (52, 44.1%)	
		Spondylosis (10, 8.5%)	
		Ligamentum flavum hypertrophy (5, 4.2%)	
		Spondylolisthesis (6, 5.1%)	
		Ossification of the posterior longitudinal ligament (3, 2.5%)	
		Spinal osteophytosis (0, 0.0%)	
		Other vertebral column degenerative disorders (3, 2.5%)	
	Vascular disorders (38, 7.9%)	Ischaemia (27, 71.1%)	
		Hemorrhage (Epidural haematoma) (9, 23.7%)	
		Vascular malformations (AV fistula, AVM) (2, 5.3%)	
	Inflammatory and autoimmune diseases (148, 30.6%)	Demyelination (142, 95.9%)	Multiple sclerosis (121, 85.2%)
			Transverse Myelitis (18, 12.7%)
			Neuromyelitis Optica (3, 2.1%)
		Collagen vascular disease (SLE, Sogren’s, RA, AS, Vasculitis) (0, 0.0%)	
		Sarcoidosis (0, 0.0%)	
		Paraneoplastic (0, 0.0%)	
		Arachnoiditis (1, 0.7%)	
		Others (5, 3.4%)	
	Neoplastic (84, 17.4%),	Benign (49, 58.3%)	Primary vertebral lesions (Osteoma, Osteochondroma, Osteoid osteoma, Haemangioma, Aneurysmal bone cyst) (0, 0.0%)
			Extradural space (Lipoma) (1, 2.0%)
			Intradural/Extramedullary (Neurofibroma, Meningioma, Schwannomas, Chordoma-benign) (40, 81.6%)
			Intramedullary (Astrocytoma-benign, Oligodendroglioma, Ependymoma, Cavernoma) (5, 10.2%)
			Other benign (3, 6.1%)
		Malignant (35, 41.7%)	Neural (Chordoma-malignant, Astrocytoma-malignant) (6, 17.1%)
			Primary vertebral lesions (Osteosarcoma, etc.) (1, 2.8%)
			Secondary vertebral lesions (Breast, Bronchus, Lung, Prostate, Renal, Thyroid, Ewing’s sarcoma, Melanoma, etc.) (18, 51.4%)
			Hematological (Myeloma, Leukemia, Non-Hodgkins Lymphoma, Hodgkin’s lymphoma) (5, 14.3%)
			Other malignant (5, 14.3%)
	Infection (83, 17.2%),	Viral (4, 4.8%)	Herpes group (Herpes simplex, Herpes zoster, CMV, Epstein–Barr), 3
			Retrovirus (HIV, HTLV-1), 0
			Enterovirus (Poliovirus, Coxsackievirus, etc.), 0
			Viral- others, 1
		Bacterial (68, 81.9%)	Staph aureus [Extradural abscess] (46, 67.6%)
			Streptococcal [Extradural abscess] (7, 10.3%)
			Mycobacterium TB (1, 1.4%)
			Brucellosis (0, 0.0%)
			Spirochaetal- *Treponema pallidum* (0, 0.0%)
			Bacterial- others (n: 14, 20.6%)
		Fungal (Cryptococcal, Actinomycosis, etc.) (1, 1.2%)	
		Parasitic (Cysticercosis, Hydatid, Toxoplasmosis, Schistosomiasis, etc.) (0, 0.0%)	
		Infections- unknown (10, 12.1%)	
	Metabolic disorders (1, 0.2%),	Deficiencies (Vitamin B12, Folate, Copper, Vitamin D), 1	
		Osteoporosis, 0	
		Paget’s disease, 0	
		Osteomalacia, 0	
		Other metabolic disorders, 0	
	Toxic (0, 0.0%)		
	Radiation-related myelitis (0, 0.0%)		
	Miscellaneous (11, 2.2%)		
Congenital or genetic disorders (4, 0.8%)	Spinal dysraphism (spina bifida occulta, myelomeningocoele, tethered cord syndrome), 2		
	Arnold-Chiari malformation, 2		
	Skeletal malformations, 0		
	Hereditary spastic paraplegia, 0		
	Spino-cerebellar, adreno-myeloneuropathy, 0		
	Spinal muscular atrophies, 0		
	Other leukodystrophies, 0		

^#^The total number of cases is higher due to common categories appearing in multiple cases.

#### SCI characteristics

**Figure 4:**
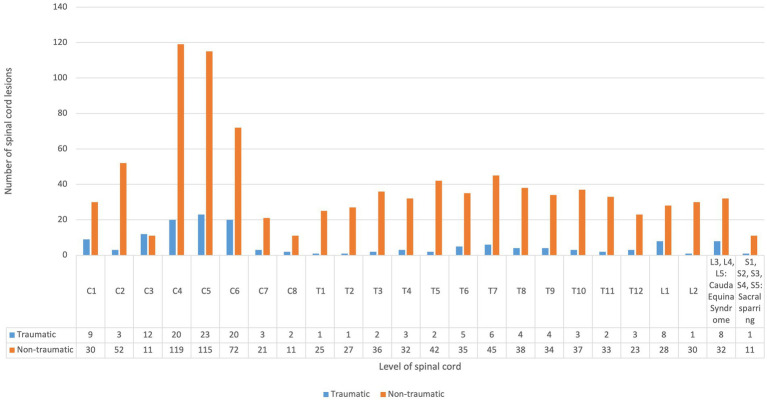
Distribution of spinal cord lesions according to the traumatic and non-traumatic cause of injury.

**Figure 5:**
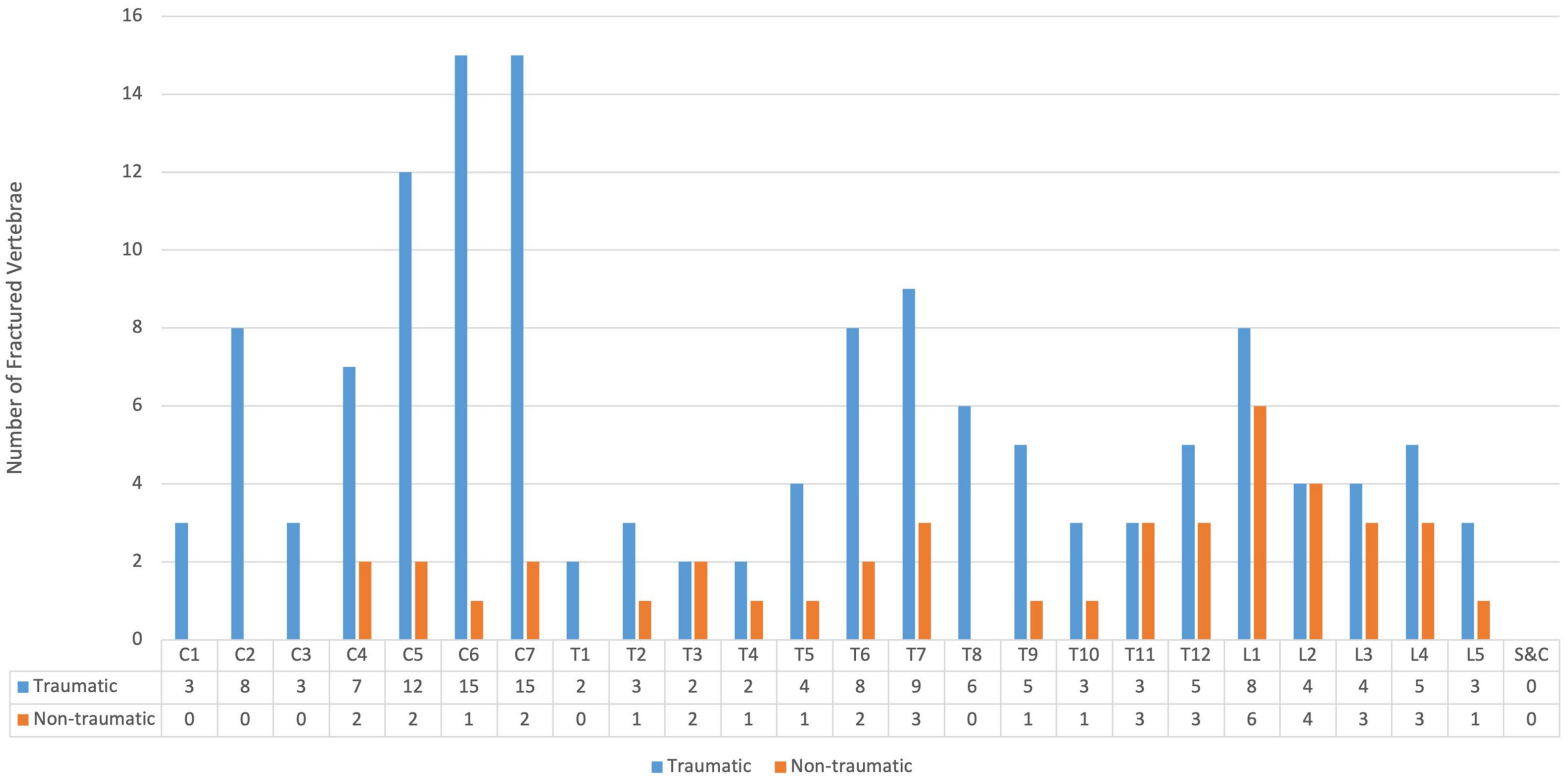
Distribution of vertebral fractures according to the traumatic and non-traumatic cause of spinal cord injury (S&C: sacral and coccygeal).

Figure 4 shows lesion distribution by cause of SCI. In total, 1,085 lesion levels were identified, including 939 in non-traumatic and 146 in traumatic cases, with many patients (especially non-traumatic) having multiple levels affected. Overall, 48.2% of lesions were cervical, 40.8% thoracic, 6.1% lumbar (including 67 conus medullaris), 3.7% cauda equina, and 1.1% sacral. The lower cervical region (C5–C8) was the most frequently involved in both non-traumatic (23.3%) and traumatic (32.9%) cases (Table 3). A total of 181 vertebral fractures were recorded, predominantly in traumatic cases (76.8%). The C5–C7 region accounted for the highest proportion (30.2%) (Figure 5). Regarding completeness, 37 SCIs were complete and 522 incomplete (data available for 559/587 cases), with traumatic cases representing 59.4% of complete injuries. Among 358 cases with available classification, paraparesis (43.3%) and tetraparesis (30.4%) were most common, followed by paraplegia (12.9%) and tetraplegia (5.9%). Less frequent presentations included hemiparesis (6.7%) and hemiplegia (0.5%). Nineteen SCI syndromes were identified: 10 central cord, 5 anterior cord, and 4 Brown-Séquard; no posterior cord syndromes were observed.

**Table 3:** Distribution of lesion levels and the cause of spinal cord injury (traumatic vs. non-traumatic).

	Upper cervical C1–C4				Lower cervical C5–C8				Upper thoracic T1–T6						Lower thoracic T7–T12						Lumbar (conus medullaris) L1–L2		Cauda equina syndrome L3–L5	Sacral sparring S1–S5
	C1	C2	C3	C4	C5	C6	C7	C8	T1	T2	T3	T4	T5	T6	T7	T8	T9	T10	T11	T12	L1	L2	L3, L4, L5	S1, S2, S3, S4, S5
Traumatic	9	3	12	20	23	20	3	2	1	1	2	3	2	5	6	4	4	3	2	3	8	1	8	1
Non-traumatic	30	52	11	119	115	72	21	11	25	27	36	32	42	35	45	38	34	37	33	23	28	30	32	11
Total	256				267				211						232						67		40	12
	523								443												107			

### Transport and hospitalization data

Among traumatic cases, hospital transport was by Emergency Medical Service (EMS) in 35 patients (43.7%), helicopter in 44 (55.0%), and private vehicle in 1 (1.2%) (data available for 80/99 cases). Most admissions occurred at CDK (415 cases, 70.6%), followed by AUVA (109 cases, 18.5%), and LKH (64 cases, 10.9%). Patients were primarily admitted to neurology (45.7%), neurosurgery (38.7%), or orthopedic surgery (12.8%) departments; one patient (0.2%) died in the emergency department. ASIA scores were documented in 49 traumatic cases: A (28.6%), B (12.2%), C (12.2%), D (40.1%), and E (6.1%). Glasgow Coma Scale (GCS) was recorded in 33 cases, with 5 scoring 3–8, 2 scoring 9–12, and 26 scoring 13–15. Early complications included CSF leakage (*n* = 4), need for mechanical ventilation (*n* = 15), fever (*n* = 26), and spasticity (*n* = 53). The most common comorbidities were hypertension and arrhythmia (cardiovascular), smoking and COPD (pulmonary), as well as diabetes and depression (other conditions) (Table 4).

**Table 4:** Medical history extracted from our cases (More than one condition might be detected in a case).

Cardiovascular diseases, 284	CVA (Cerebrovascular accident), 32
	MI (Miocardial Infarction), 12
	Peripheral vascular disease, 15
	Hypertension, 223
	Hypotension, 1
	CHF (Chronic Heart Failiure), 6
	CAD (Coronary artery disease), 6
	DVT (Deep vein thrombosis), 26
	Arrhythmias, 52
	Cardiac Pacemaker, 6
	Cardiac surgery, 11
	Hypercholesterolemia, 12
	Other cardiac disorders, 44
Pulmonary disease, 124	Asthma, 15
	COPD, 38
	Sleep apnea, 11
	Smoking history, 58
Other medical conditions, 402	Diabetes Mellitus, 83
	ESRD, 4
	Chronic kidney disease, 5
	Cirrhosis, 4
	Dementia, 3
	AIDS, 0
	Rheumatologic diseases, 27
	Epilepsy, 24
	Depression, 40
	Malignancy (other than spine), 6
	Thyroid dysfunction, 5
	Others, 293

### Interventions and patients’ outcome

In total, 334 patients underwent surgical intervention, including 123 spinal fixations and fusions (25 single-level, 37 two-level, 50 multi-level, and 11 unspecified). Non-surgical management was documented in 113 cases, mainly external immobilization (*n* = 71) or enforced bed rest (*n* = 42). Corticosteroids were administered at admission in 134 patients—20 of 99 traumatic cases (20.2%) and 114 of 488 non-traumatic cases (23.3%). Bladder incontinence or retention was documented in 174 cases (29.6%), while bowel incontinence occurred in 50 cases (8.5%). The total ICU length of stay was 1,514 days (1,025 for non-traumatic and 488 for traumatic cases). Median ICU stay was 2.1 days for non-traumatic and 4.9 days for traumatic cases. Table 5 summarizes patient status at first hospital discharge. According to Statistik Austria, 138 deaths (24%) were recorded up to January 2025, including 23 traumatic and 115 non-traumatic cases. Further analyses of mortality, including crude and standardized mortality ratios as well as short- and long-term survival, will be reported separately.

**Table 5:** Condition at the time of first discharge from the hospital (data available for 518 out of 587 cases, 88.2%).

Good recovery	Resumption of normal life with the capacity to work even if pre-injury status has not been achieved. Some patients have minor neurological or psychological deficits	255, 49.2%
Moderate disability	Patients have some disability such as aphasia, hemiparesis or epilepsy and/or deficits of memory or personality but are able to look after themselves. They are independent at home but dependent outside	132, 25.5%
Severe disability	Patients are dependent to daily support for mental and/or physical disability	107, 20.6%
Unresponsive wakefulness or vegetative state	Condition of unawareness with only reflexive responses but with periods of spontaneous eye opening	1, 0.1%
Deceased		23, 4.4%

## Discussion

The incidence of SCI for Salzburg citizens from 2013 to 2023 was 9.7 per 100.000/year including both traumatic and non-traumatic cases with significantly more in non-traumatic cases (1.6 vs. 8.1 per 100.000/year). The male-to-female ratio of 2.96 in traumatic cases and 1.02 in non-traumatic cases in our study indicates that males are significantly more vulnerable to traumatic injuries. The median ages of traumatic and non-traumatic cases were similar, although a younger age profile for traumatic cases would have been expected based on previous literature (16, 17). In a population-based study in Canada for example, non-traumatic SCI occurred increasingly commonly in the older population, as compared to traumatic SCI with a peak in prevalence around age 50 (18). This may be explained by the high number of falls among older patients in our study, which were the leading cause of traumatic SCI. From 2020 onward, we observed a sharp decline in both non-traumatic and traumatic cases, falling from an average of 50 non-traumatic and 11 traumatic cases per year to much lower numbers. This trend temporally coincides with the onset of the COVID-19 pandemic and is likely multifactorial, potentially reflecting reduced physical activity, fewer accidents, limited hospital access, diagnostic delays, underreporting during healthcare system strain, and patients’ reluctance to seek medical care.

Interestingly, this trend remained stable after the pandemic and may be attributed to sustained changes in behavior, but possibly also earlier identification of and better treatment for non-traumatic cases (12, 13, 19). About half of the injuries in our study were cervical, 40% thoracic, and around 10% lumbar. In the traumatic group, more than 60% were cervical, with the lower cervical region (C5–C8) most frequently affected in both traumatic and non-traumatic cases. Vertebral fractures were predominantly traumatic, with an average of 1.4 fractures per case compared to 0.08 in non-traumatic cases. Approximately one quarter of patients in both groups received corticosteroids at admission. ICU stays were significantly longer in traumatic cases, underscoring their greater impact on healthcare costs and overall disease burden. The leading causes of traumatic spinal cord injury in our study were falls, transport accidents, and sports-related injuries. Falls and transport injuries are the primary causes of traumatic SCI globally (2). During the study period, 65 sports-related SCIs occurred in Salzburg, of which 19 involved Salzburg residents and were included in our incidence analysis. Considering all cases in the Federal State of Salzburg (residents and non-residents), sports injuries were dominated by skiing (32 cases, 50.8%), mountain/downhill biking (20 cases), and paragliding (6 cases). Among Salzburg residents, only 7 ski accidents, 4 biking accidents, and 2 paragliding accidents were recorded. Notably, 25% of traumatic SCIs in Salzburg affected non-Austrian nationals, reflecting the impact of sports tourism, particularly skiing, whereas only 8% of non-traumatic cases were non-Austrian. Overall, 30% of traumatic SCIs occurred in sports facilities, and 65% were related to sports or leisure activities. Skiing and mountain biking together accounted for over 80% of sports-related SCIs, underlining the vulnerability of Salzburg as a major tourist destination. Falls and transport accidents were also prominent causes, emphasizing the need for preventive measures such as fall-prevention programs and stricter transport safety regulations. Lack of protective equipment contributed to injury severity—20 traumatic cases had no safety device in use, while helmets were documented in 19 cases. Information on seat belts, airbags, child seats, and protective clothing was incomplete. Nearly 40% of traumatic SCIs occurred on weekends, highlighting the role of leisure activities, while most weekday cases may be work-related. Seasonally, injuries peaked in September, with additional increases in summer months, likely reflecting higher outdoor activity and tourism.

Multiple sclerosis and related disorders were the most common cause of non-traumatic SCI in our cohort, accounting for 121 cases. Among 938 MS patients screened, only 12.9% had spinal lesions and were classified as non-traumatic SCI. Many had multiple lesion levels, with 193 cervical lesions in 106 cases and 62 thoracic lesions in 42 cases. Women were more frequently affected (64.4%). Similar findings were observed in spinal stenosis, where 61 of 885 screened cases (6.8%) developed myelopathy. Noteworthy is that clinical presentation in non-traumatic SCI, particularly inflammatory etiologies such as multiple sclerosis, may be progressive or fluctuating and is not always captured by acute neurological severity scales. Approximately half of the non-traumatic cases in our study exhibited a prolonged symptom onset, with symptoms persisting for more than 1 month, while about 25% had a symptom duration of 7 days to 1 month. This highlights the more chronic nature of non-traumatic SCIs compared to traumatic cases. Interestingly, among the 50 cases of acute presentation in the non-traumatic group, 64% (32 cases) were categorized under vascular disorders, 16% (8 cases) were associated with demyelinating disorders (multiple sclerosis), and 8% (4 cases) were attributed to infections, which may contribute to the nature and characteristics of these conditions.

For all SCI cases, we used the registered home address as a proxy for residence, in addition to the location of injury. This approach allowed us to capture cases directly relevant to Salzburg County. Consequently, cases in which the injury occurred outside the county were excluded, even if patients were transferred to Salzburg hospitals (SALK or AUVA). From a healthcare perspective, however, these cases still impose a considerable burden. Of the 195 traumatic SCIs recorded at AUVA, nearly half (47.6%) originated outside Salzburg County but were treated at the regional trauma center. Similarly, many SALK admissions involved transfers or patients with injuries predating 2013, which were excluded from analysis but nonetheless contribute significantly to healthcare demand. Such cases highlight the ongoing clinical and economic impact of SCI, as treatment and rehabilitation often extend for years or even lifelong.

While some data on the incidence and mortality of traumatic SCI exist for Austria (6), non-traumatic SCI has not been systematically reported and remains underrepresented internationally, with only a few country-level studies available (11, 12). In high-income European settings, reported SCI incidence rates vary substantially depending on case definitions and whether non-traumatic etiologies are included. Population-based and rehabilitation-based cohorts from Switzerland have reported non-traumatic SCI incidence rates comparable to those observed in Salzburg, particularly in aging populations with a high burden of degenerative and inflammatory spinal disorders (18). In this context, the Salzburg data align closely with other European healthcare systems characterized by universal coverage and centralized specialist care. Most SCI research has focused on traumatic cases, which are easier to record, whereas non-traumatic SCI poses greater challenges due to its heterogeneous causes, including infections, inflammation, and degenerative disorders. The huge difference between our incidence data (9.7 per 100,000) with the previous Austrian study (1.39 per 100,000) comes from non-traumatic cases which contained more than 80% of our cases and were not included in the previous study (6). SCI incidence rates vary globally, influenced by factors such as regional reporting practices and healthcare infrastructure. While global incidence estimates vary substantially, particularly in low- and middle-income settings (1, 20), comparisons with European cohorts are more relevant for Austria. In this regard, the traumatic SCI incidence in Salzburg lies at the lower end of the range reported across Europe, whereas non-traumatic SCI rates are similar to those observed in Switzerland and other high-income countries (18, 23). Using data from GBD collaboration, the researchers of this project have recently published a paper, systematically assessing global, regional, and national trends of SCI (2). This study revealed that despite small changes in age-standardized rates, the absolute numbers of cases and disability have increased substantially, with notable variations across demographics and regions (24). These variations underscore the need for standardized reporting and comprehensive data collection to accurately assess SCI’s global impact. An understanding of the incidence and causes of SCI is essential for planning cost-effective care and for developing preventive strategies (22).

Compared internationally, the traumatic SCI incidence in Salzburg lies at the lower end of the global range (0.36–19.5 per 100,000), while our non-traumatic rates are similar to those reported in Switzerland (1.8 per 100,000/year) and Australia (2.6 per 100,000/year) (18, 21).

By restricting incidence estimates to Salzburg residents, we differentiated between overall case burden (including tourists) and true hospital network–based, population-referenced incidence. This distinction is relevant in Salzburg, where a high proportion of traumatic cases—especially skiing and mountain biking accidents—involve non-Austrian tourists. While excluded from incidence calculations, such cases remain important for healthcare system planning. Overall, our findings demonstrate that non-traumatic SCI represents the majority of cases in Salzburg, contrasting with the global research focus on traumatic SCI. Preventive strategies should therefore target not only fall and transport injuries but also degenerative and inflammatory conditions. Establishing a prospective SCI registry in Austria, covering both traumatic and non-traumatic etiologies, would provide more reliable data for health policy and long-term planning.

## Limitations of the study

This study has several limitations inherent to its retrospective design. Data completeness depended on routine clinical documentation, resulting in missing information for some variables, particularly prehospital details and socioeconomic factors such as educational level. Long-term functional outcomes, quality of life, and psychosocial consequences could not be assessed. Case ascertainment was based on a regional hospital network rather than a population registry. Although this network captures the majority of clinically relevant SCI cases in Salzburg, under-ascertainment remains possible, particularly for residents treated entirely outside the network or managed through outpatient-only pathways. In addition, Salzburg residents sustaining traumatic SCI outside the county and managed exclusively outside the regional hospital system may not have been captured, potentially leading to underestimation of traumatic SCI incidence. Because complete longitudinal data preceding 2013 were not universally available across all data sources, a formal incident washout period could not be applied. Incidence estimates should therefore be interpreted as first documented hospital presentations within the study period rather than definitive first-ever disease onset, particularly for chronic non-traumatic etiologies. Lesion distribution was reported at the lesion level rather than by a single index level per patient in order to reflect multilevel spinal cord involvement. This approach may overrepresent multilevel disease, especially in non-traumatic SCI, and should be interpreted accordingly. Finally, although all cases underwent independent manual review by two investigators with consensus resolution, formal inter-rater reliability statistics were not calculated, which may affect reproducibility in etiologic classification for borderline cases. Future prospective studies using standardized case definitions, complete longitudinal capture, and population-based registries would allow more precise incidence estimates and a more comprehensive assessment of long-term outcomes and healthcare burden.

## Conclusion

In Salzburg, the incidence of traumatic and non-traumatic spinal cord injury was 9.7 per 100,000/year, with non-traumatic cases accounting for more than 80%. Multiple sclerosis and related disorders were the leading non-traumatic cause, while falls were the predominant traumatic mechanism. These findings emphasize the need for preventive strategies targeting degenerative and inflammatory conditions, alongside injury prevention for falls and sports-related activities.

## Data Availability

The original contributions presented in the study are included in the article/Supplementary material, further inquiries can be directed to the corresponding author.
