# Supplementary material for: Incidence and causes of traumatic and non-traumatic spinal cord injury in Salzburg, Austria: a multi-center hospital network–based study
Source: Front Neurol. 2026 Feb 6;17:1728274. doi: 10.3389/fneur.2026.1728274 (PMC12920206; doi:10.3389/fneur.2026.1728274)
Supplement: Supplementary file 1 [file Data_Sheet_1.pdf]

| ICD 10 | Description | ICD 9 | Description |
|---|---|---|---|
| C70 | Malignant neoplasm of meninges | 192 | Malignant neoplasm of other and unspecified parts of nervous system |
| C70.0 | Malignant neoplasm of cerebral meninges | 192.1 | Malignant neoplasm of cerebral meninges |
| C70.1 | Malignant neoplasm of spinal meninges | 192.2 | Malignant neoplasm of spinal cord |
| C70.9 | Malignant neoplasm of meninges, unspecified | 192.3 | Malignant neoplasm of spinal meninges |
| C72 | Malignant neoplasm of spinal cord, cranial nerves and other parts of central nervous system | 192.8 | Malignant neoplasm of other specified sites of nervous system |
| C72.0 | Malignant neoplasm of spinal cord | 192.9 | Malignant neoplasm of nervous system, part unspecified |
| C72.1 | Malignant neoplasm of cauda equina | | |
| C72.9 | Malignant neoplasm of central nervous system, unspecified | 225 | Benign neoplasm of brain and other parts of nervous system |
| | | 225.2 | Benign neoplasm of cerebral meninges |
| | | 225.3 | Benign neoplasm of spinal cord |
| D32 | Benign neoplasm of meninges | 225.4 | Benign neoplasm of spinal meninges |
| D32.0 | Benign neoplasm of cerebral meninges | 225.8 | Benign neoplasm of other specified sites of nervous system |
| D32.1 | Benign neoplasm of spinal meninges | 225.9 | Benign neoplasm of central nervous system, part unspecified |
| D32.9 | Benign neoplasm of meninges, unspecified | | |
| D33 | Benign neoplasm of brain and other parts of central nervous system | 323 | Encephalitis myelitis and encephalomyelitis |
| D33.4 | Benign neoplasm of spinal cord | 323.1 | Encephalitis, myelitis, and encephalomyelitis in rickettsial diseases classified elsewhere |
| D33.9 | Benign neoplasm of central nervous system, unspecified | 323.2 | Encephalitis, myelitis, and encephalomyelitis in protozoal diseases classified elsewhere |
| | | 323.4 | Other encephalitis, myelitis, and encephalomyelitis due to infection classified elsewhere |
| | | 323.5 | Encephalitis, myelitis, and encephalomyelitis following immunization procedures |
| G04 | Encephalitis, myelitis and encephalomyelitis | 323.6 | Postinfectious encephalitis, myelitis, and encephalomyelitis |
| G04.0 | Acute disseminated encephalitis and encephalomyelitis (ADEM) | 323.7 | Toxic encephalitis, myelitis, and encephalomyelitis |
| G04.1 | Tropical spastic paraplegia | 323.8 | Other causes of encephalitis, myelitis, and encephalomyelitis |
| G04.2 | Bacterial meningoencephalitis and meningomyelitis, not elsewhere classified | 323.9 | Unspecified causes of encephalitis, myelitis, and encephalomyelitis |
| G04.8 | Other encephalitis, myelitis and encephalomyelitis | | |
| G04.9 | Encephalitis, myelitis and encephalomyelitis, unspecified | 324 | Intracranial and intraspinal abscess |
| G05* | Encephalitis, myelitis and encephalomyelitis in diseases classified elsewhere | 324.1 | Intraspinal abscess |
| G05.0* | Encephalitis, myelitis and encephalomyelitis in bacterial diseases classified elsewhere | 324.9 | Intracranial and intraspinal abscess of unspecified site |
| G05.1* | Encephalitis, myelitis and encephalomyelitis in viral diseases classified elsewhere | 325 | Phlebitis and thrombophlebitis of intracranial venous sinuses |
| G05.2* | Encephalitis, myelitis and encephalomyelitis in other infectious and parasitic diseases classified elsewhere | 326 | Late effects of intracranial abscess or pyogenic infection |
| G05.8* | Encephalitis, myelitis and encephalomyelitis in other diseases classified elsewhere | | |
| G06 | Intracranial and intraspinal abscess and granuloma | 335 | Anterior horn cell disease |
| G06.1 | Intraspinal abscess and granuloma | 335.10 | Spinal muscular atrophy, unspecified |
| G06.2 | Extradural and subdural abscess, unspecified | 335.19 | Other spinal muscular atrophy |
| G07* | Intracranial and intraspinal abscess and granuloma in diseases classified elsewhere | 335.2 | Motor neuron disease |
| G08 | Intracranial and intraspinal phlebitis and thrombophlebitis | 335.8 | Other anterior horn cell diseases |
| G09 | Sequelae of inflammatory diseases of central nervous system | 335.9 | Anterior horn cell disease, unspecified |
| G11.4 | Hereditary spastic paraplegia | | |
| G12 | Spinal muscular atrophy and related syndromes | 340 | Multiple sclerosis |
| G12.0 | Infantile spinal muscular atrophy, type I [Werdnig-Hoffman] | 341 | Other demyelinating diseases of central nervous system |
| G12.1 | Other inherited spinal muscular atrophy | 341.2 | Acute (transverse) myelitis |
| G12.2 | Motor neuron disease | 341.9 | Demyelinating disease of central nervous system, unspecified |
| G12.8 | Other spinal muscular atrophies and related syndromes | | |
| G12.9 | Spinal muscular atrophy, unspecified | 344 | Other paralytic syndromes |
| G35 | Multiple sclerosis | 344.0 | Quadriplegia and quadraparesis |
| G36 | Other acute disseminated demyelination | 344.1 | Paraplegia |
| G36.0 | Neuromyelitis optica [Devic] | 344.8 | Other Specified paralytic syndromes |
| G36.8 | Other specified acute disseminated demyelination | 344.2 | Diplegia of upper limbs |
| G36.9 | Acute disseminated demyelination, unspecified | 344.6 | Cauda equina syndrome |
| G37.3 | Acute transverse myelitis in demyelinating disease of central nervous system | 344.9 | paralysis, unspecified |
| G37.4 | Subacute necrotizing myelitis | 336 | Other diseases of spinal cord |
| G82 | Paraplegia and tetraplegia | 336.0 | Syringomyelia and syringobulbia |
| G82.0 | Flaccid paraplegia | 336.1 | Vascular myelopathies |
| G82.1 | Spastic paraplegia | 336.8 | Other myelopathy |
| G82.2 | Paraplegia, unspecified | 336.9 | Unspecified disease of spinal cord |
| G82.3 | Flaccid tetraplegia | 336.3 | Myelopathy in other diseases classified elsewhere |
| G82.4 | Spastic tetraplegia | 336.2 | Subacute combined degeneration of spinal cord i n diseases classified elsewhere |
| G82.5 | Tetraplegia, unspecified | | |
| G83 | Other paralytic syndromes | 433.2 | Occlusion and stenosis of vertebral artery |
| G83.0 | Diplegia of upper limbs | | |
| G83.4 | Cauda equina syndrome | 721 | Spondylosis |
| G95 | Other diseases of spinal cord | 721.1 | Cervical spondylosis with myelopathy |
| G95.0 | Syringomyelia and syringobulbia | 721.4 | Thoracic or lumbar spondylosis with myelopathy |
| G95.1 | Vascular myelopathies | 721.8 | Other allied disorders of spine |
| G95.2 | Cord compression, unspecified | 721.9 | Spondylosis of unspecified site |
| G95.8 | Other specified diseases of spinal cord | 722 | Intervertebral disc disorders |
| G95.9 | Disease of spinal cord, unspecified | 722.11 | Disc herniation |
| G99.2 | Myelopathy in diseases classified elsewhere | 722.5 | Disc degeneration |
| G99.8 | Other specified disorders of nervous system in diseases classified elsewhere | 722.7 | Intervertebral disc disorder with myelopathy |
| | | 722.9 | Other and unspecified disc disorder |
| M46.20 | Osteomyelitis of vertebra, site unspecified | 722.92 | Infection, disc space |
| M47.0 | Anterior spinal and vertebral artery compression syndromes | 722.93 | Disc space infection |
| M47.1 | Other spondylosis with myelopathy | 723.0 | Spinal stenosis in cervical region |
| M47 | Spondylosis, unspecified | 724 | Spinal stenosis |
| M48.0 | Spinal stenosis | 730.28 | Infection, vertebral |
| M48.9 | Spondylopathy, unspecified | 733.13 | Fracture, compression, spontaneous |
| M48.50 | Collapsed vertebra, not elsewhere classified, site unspecified, initial encounter for fracture | | |
| M49* | Spondylopathies in diseases classified elsewhere | 0.15.0 | Tuberculosis of vertebral column |
| M49.0 | Tuberculosis of spine | 0.23 | Brucellosis |
| M49.1 | Brucella spondylitis | | |
| M94.4 | Neuropathic spondylopathy | | |
| M49.5 | Collapsed vertebra in diseases classified elsewhere | 805 | Fracture |
| M50 | Cervical disc disorders | 805.6 | Fracture-coccyx |
| M50.0 | Cervical disc disorder with myelopathy, unspecified cervical region | 806 | Fracture of vertebral column with spinal cord injury |
| M50.20 | Other cervical disc displacement, unspecified cervical region | 839.21 | Dislocation, vertebral |
| M50.80 | Other cervical disc disorders, unspecified cervical region | 847.1 | Sprain |
| M50.90 | Cervical disc disorder, unspecified, unspecified cervical region | 847.3 | Strain, non traumatic musculoskeletal |
| M51 | Other intervertebral disc disorders | | |
| M51.0 | Thoracic, thoracolumbar and lumbosacral intervertebral disc disorders with myolopathy | 925.2 | Crushing injury of neck |
| | | 952 | Spinal cord injury without evidence of spinal bone injury |
| M80.08 | Age-related osteoporosis with current pathological fracture, vertebra(e), initial encounter for fracture | | |
| M84.48 | Pathological fracture, other site, initial encounter for fracture | 952.4 | Cauda equina spinal cord injury without evidence of spinal bone injury |
| M84.68 | Pathological fracture in other disease, other site, initial encounter for fracture | 953 | Injury to nerve roots and spinal plexus |
| M99.2 | Subluxation stenosis of neural canal | 953.5 | Injury to lumbosacral plexus |
| M99.3 | Osseous stenosis of neural canal | 968 | Spinal cord lesion without evidence of spine injury |
| M99.4 | Connective tissue stenosis of neural canal | 996.2 | Mechanical complication of nervous system device, implant, and graft |
| M99.5 | Intervertebral disc stenosis of neural canal | | |
| | | | |
| Q05.9 | Spina bifida, unspecified | | |
| S12 | Fracture of neck | | |

| | |
|---|---|
| **S12.0** | Fracture of first cervical vertebrae |
| **S12.1** | Fracture of second cervical vertebrae |
| **S12.2** | Fracture of third cervical vertebrae |
| **S12.3** | Fracture of fourth cervical vertebrae |
| **S12.4** | Fracture of fifth cervical vertebrae |
| **S12.5** | Fracture of sixth cervical vertebrae |
| **S12.6** | Fracture of seventh cervical vertebrae |
| **S12.7** | Multiple fractures of cervical spine |
| **S12.9** | Fracture of neck, unspecified, initial encounter |
| **S13.0** | Traumatic rupture of cervical intervertebral disc |
| **S13.101** | Dislocation of unspecified cervical vertebrae, initial encounter |
| **S13.2** | Dislocation of other and unspecified parts of neck |
| **S13.4** | Sprain and strain of cervical spine |
| **S13.8** | Sprain of joints and ligaments of other parts of neck, initial encounter |
| **S14** | Injury of nerves and spinal cord at neck level |
| **S14.0** | Concussion and edema of cervical spinal cord |
| **S14.1** | Other and unspecified injuries of cervical spinal cord |
| **S22.0** | Fracture of thoracic vertebrae |
| **S23.0** | Traumatic rupture of thoracic intervertebral disc |
| **S23.1** | Dislocation of thoracic vertebrae |
| **S24** | Injury of nerves and spinal cord at thorax level |
| **S24.0** | Concussion and edema of thoracic spinal cord |
| **S24.1** | Other and unspecified injuries of thoracic spinal cord |
| **S32.0** | Fracture of lumbar vertebrae |
| **S32.1** | Unspecified fracture of sacrum, initial encounter for closed fracture |
| **S32.2** | Fracture of coccyx, initial encounter for closed fracture |
| **S33.0** | Traumatic vertebrae of lumbar intervertebral disc |
| **S33.1** | Dislocation of lumbar vertebrae |
| **S33.5** | Sprain of ligaments of lumbar spine, initial encounter |
| **S34** | Injury of nerves and lumbar spinal cord at abdomen, lower back and pelvis level |
| **S34.0** | Concussion and oedema of lumbar spinal cord |
| **S34.1** | Concussion and edema of lumbar and sacral spinal cord |
| **S34.2** | Other and unspecified injury of lumbar or sacral spinal cord |
| **S34.3** | Injury of cauda equina |
| **S34.4** | Injury of lumbosacral plexus |
| | |
| **T06.0** | Injury of brain and cranial nerves with injuries of nerves and spinal cord at neck level |
| **T06.1** | Injuries of nerves and spinal cord involving other multiple body regions |
| **T09.3** | Injury of spinal cord, level unspecified |
| **T85.1** | Mechanical complication of implanted electronic stimulator of nervous system |
| **T91** | Sequelae of injuries, of poisoning and of other consequences of external causes- Sequelae of injuries of neck and trunk |
| **T91.1** | Sequelae of injuries, of poisoning and of other consequences of external causes- Sequelae of injuries of neck and trunk – Sequelae of fracture of spine |
| **T91.3** | Sequelae of injuries, of poisoning and of other consequences of external causes- Sequelae of injuries of neck and trunk – Sequelae of injury of spinal cord |

*Data Dictionary Codebook*

# Epidemiologic studies of Spinal Cord Injury: Traumatic and non-Traumatic (PID: 126)

03.14.2025 17:40

| **Instruments** | |
|---|---|
| Instrument | Form Name |
| My First Instrument | my_first_instrument |

| | # | Variable / Field Name | Field Label *Field Note* | Field Attributes (Field Type, Validation, Choices, Calculations, etc.) |
|---|---|---|---|---|
| **Instrument: My First Instrument (my_first_instrument)** | | | | |
| | 1 | [record_id] | Record number | text |
| | 2 | [first_name] | First Name | text, Required, Identifier |
| | 3 | [last_name] | Last Name | text, Required, Identifier |
| | 4 | [patient_id] | Patient ID | text |
| | 5 | [gender] | Sex | radio, Required<br>1 Male<br>2 Female<br>3 Others |
| | 6 | [birth_date] | Birth date | text (date_dmy) |
| | 7 | [nationality] | Nationality | dropdown, Identifier<br>1 Afghan<br>2 Albanian<br>3 Algerian<br>4 Argentine<br>5 Argentinian<br>6 Australian<br>7 Austrian |

| | |
|---|---|
| 8 | Azerbaijani |
| 9 | Bangladeshi |
| 10 | Belgian |
| 11 | Bolivian |
| 12 | Batswana |
| 13 | Bosnian |
| 14 | Brazilian |
| 15 | Bulgarian |
| 16 | Cambodian |
| 17 | Cameroonian |
| 18 | Canadian |
| 19 | Chilean |
| 20 | Chinese |
| 21 | Colombian |
| 22 | Costa Rican |
| 23 | Croatian |
| 24 | Cuban |
| 25 | Czech |
| 26 | Danish |
| 27 | Dominican |
| 28 | Dutch |
| 29 | Ecuadorian |
| 30 | Egyptian |
| 31 | Emirati |
| 32 | English |
| 33 | Estonian |
| 34 | Ethiopian |

| | |
|---|---|
| 35 | Fijian |
| 36 | Finnish |
| 37 | French |
| 38 | German |
| 39 | Ghanaian |
| 40 | Greek |
| 41 | Guatemalan |
| 42 | Haitian |
| 43 | Honduran |
| 44 | Hungarian |
| 45 | Icelandic |
| 46 | Indian |
| 47 | Indonesian |
| 48 | Iranian |
| 49 | Iraqi |
| 50 | Irish |
| 51 | Israeli |
| 52 | Italian |
| 53 | Jamaican |
| 54 | Japanese |
| 55 | Jordanian |
| 56 | Kenyan |
| 57 | Kosovan |
| 58 | Kuwaiti |
| 59 | Lao |
| 60 | Latvian |
| 61 | Lebanese |

| | |
|---|---|
| 62 | Libyan |
| 63 | Lithuanian |
| 64 | Macedonian |
| 65 | Malagasy |
| 66 | Malaysian |
| 67 | Malian |
| 68 | Maltese |
| 69 | Mexican |
| 70 | Mongolian |
| 71 | Moroccan |
| 72 | Mozambican |
| 73 | Namibian |
| 74 | Nepalese |
| 75 | New Zealander |
| 76 | Nicaraguan |
| 77 | Nigerian |
| 78 | Norwegian |
| 79 | Pakistani |
| 80 | Panamanian |
| 81 | Paraguayan |
| 82 | Peruvian |
| 83 | Philippine |
| 84 | Polish |
| 85 | Portuguese |
| 86 | Romanian |
| 87 | Russian |
| 88 | Salvadoran |

| | |
|---|---|
| 89 | Saudi |
| 90 | Scottish |
| 91 | Senegalese |
| 92 | Serbian |
| 93 | Singaporean |
| 94 | Slovak |
| 95 | Somali |
| 96 | South African |
| 97 | South Korean |
| 98 | Spanish |
| 99 | Sri Lankan |
| 100 | Sudanese |
| 101 | Swedish |
| 102 | Swiss |
| 103 | Syrian |
| 104 | Taiwanese |
| 105 | Tajikistani |
| 106 | Thai |
| 107 | Tongan |
| 108 | Tunisian |
| 109 | Turkish |
| 110 | Ukrainian |
| 111 | Uruguayan |
| 112 | Venezuelan |
| 113 | Vietnamese |
| 114 | Welsh |
| 115 | Zambian |

| | | | | |
|---|---|---|---|---|
| | | | | 116 Zimbabwean |
| | 8 | [marital_status_at_the_time] | Marital status (at the time of injury) | radio<br>1 Single<br>2 Married<br>3 Widow(er)<br>4 Divorced<br>5 Unknown |
| | 9 | [education_level] | Education level | radio<br>1 None (illiterate)<br>2 Compulsory schooling (9 years)<br>3 High school (9-12 years)<br>4 College degree (Bachelor and Master)<br>5 Graduate degree (Doctorate) and higher<br>6 Unknown |
| | 10 | [telephone_number_1_includi] | Telephone number (Including country code) | text, Required |
| | 11 | [address] | Address | text |
| | 12 | [city_of_residence] | City of residence at the time of injury | text, Identifier |
| | 13 | [city_where_the_injury_happ] | City where the injury happened | text, Required |
| | 14 | [postal_code] | Postal code | text |
| | 15 | [type_of_injury] | Type of Injury | radio, Required, Identifier<br>1 Traumatic<br>2 Non-traumatic |
| | 16 | [traumatic]<br>Show the field ONLY if:<br>[type_of_injury] = '1' | Traumatic cause | radio<br>1 Transport injuries<br>2 Falls<br>3 Sports |

| | | | | |
|---|---|---|---|---|
| | | | | 4 Drowning<br>5 Fire and hot substances<br>6 Conflict, assault or violence<br>7 Gunshot wounds<br>8 Suicide<br>9 Disasters<br>11 Occupational<br>10 Others |
| | 17 | [transport_injuries_type]<br>Show the field ONLY if:<br>[traumatic] = '1' | Transport injuries type | radio<br>1 Motor Vehicle Crash (Car accident)<br>2 Pedestrian<br>3 Cyclist<br>4 Motorcyclist<br>5 Heavy vehicles<br>6 Bus<br>7 Train |
| | 18 | [type_of_sport]<br>Show the field ONLY if:<br>[traumatic] = '3' | Type of sport | radio<br>1 Ski<br>2 Diving<br>3 Football (Soccer)<br>4 Hockey<br>5 Hiking<br>6 Rock climbing<br>7 Auto racing<br>8 Motorbike racing<br>9 Mountain or downhill biking |

| | | | | |
|---|---|---|---|---|
| | | | | 10 Paragliding<br>11 Others |
| | 19 | [injury_time]<br><br>Show the field ONLY if:<br>[timeframe_of_clinical_symp] = '1' or [type_of_injury] = '1' | Injury date | text (date_dmy) |
| | 20 | [injury_time_hour]<br><br>Show the field ONLY if:<br>[timeframe_of_clinical_symp] = '1' or [type_of_injury] = '1' | Injury time | text (time) |
| | 21 | [day_of_injury]<br><br>Show the field ONLY if:<br>[type_of_injury] = '1' | Day of injury | radio<br>1 Monday<br>2 Tuesday<br>3 Wednesday<br>4 Thursday<br>5 Friday<br>6 Saturday<br>7 Sunday |
| | 22 | [activity_when_the_injury_h]<br><br>Show the field ONLY if:<br>[type_of_injury] = '1' | Activity when the injury happened | radio<br>1 Sports activity<br>2 Leisure activity<br>3 Working for income activity<br>4 Other activities (resting, sleeping, eating, or engaging)<br>5 Unspecified activities |
| | 23 | [location_of_injury]<br><br>Show the field ONLY if:<br>[type_of_injury] = '1' | Place where injury happened | radio<br>1 Home<br>2 Residential institution |

| | | | | |
|---|---|---|---|---|
| | | | | 3 Sports and athletics area<br>4 School, other institution and public administrative area<br>5 Street and highway<br>6 Trade and service area<br>7 Industrial and construction area<br>8 Farming land<br>9 Other specified places<br>10 Unspecified place |
| | 24 | [mechanism_of_injury]<br><br>Show the field ONLY if:<br>[type_of_injury] = '1' | Mechanism of injury | radio<br>1 Penetrating<br>2 Blunt<br>3 Undetermined |
| | 25 | [safety_devices_at_the_time]<br><br>Show the field ONLY if:<br>[type_of_injury] = '1' | Safety devices at the time of injury | radio<br>1 Helmet<br>2 Seat belt<br>3 Airbag<br>7 Protective clothing<br>4 Child seat<br>5 None<br>6 Unknown |
| | 26 | [non_traumatic]<br><br>Show the field ONLY if:<br>[type_of_injury] = '2' | Non-traumatic cause | radio<br>1 Congenital OR Genetic disorders<br>2 Aquired abnormalities |
| | 27 | [congenital_or_genetic_diso]<br><br>Show the field ONLY if:<br>[non_traumatic] = '1' | Congenital OR Genetic disorders | radio |

| | | | | |
|---|---|---|---|---|
| | | | | 1 Spinal dysraphism (Spina bifida occulta, Myelomeningocoele, Tethered cord syndrome, Cauda equina syndrome)<br>2 Arnold-Chiari malformation<br>3 Skeletal malformations<br>4 Hereditary spastic paraplegia<br>5 Spino-cerebellar<br>6 Adreno-myeloneuropathy<br>7 Other leukodystrophies<br>8 Spinal muscular atrophies<br>9 Other |
| | 28 | [aquired_abnormalitites]<br>Show the field ONLY if:<br>[non_traumatic] = '2' | Aquired abnormalitites | radio<br>1 Vertebral column degenerative disorders<br>2 Metabolic disorders<br>3 Vascular disorders<br>4 Inflammatory and auto-immune diseases<br>5 Radiation-related myelitis<br>6 Toxic<br>7 Neoplastic<br>8 Infection<br>9 Miscellaneous |
| | 29 | [vertebral_column_degenerat]<br>Show the field ONLY if:<br>[aquired_abnormalitites] = '1' | Vertebral column degenerative disorders | checkbox<br>1 vertebral_column_degenerat___1 Disc prolapse<br>2 vertebral_column_degenerat___2 Ligamentum flavum hypertrophy<br>3 vertebral_column_degenerat___3 Ossification of the posterior |

| | | | | |
|---|---|---|---|---|
| | | | | (continued) longitudinal ligament<br>4 vertebral_column_degenerat___4 Spinal osteophytosis<br>5 vertebral_column_degenerat___5 Spondylolisthesis<br>6 vertebral_column_degenerat___6 Spondylosis<br>7 vertebral_column_degenerat___7 Spinal stenosis<br>8 vertebral_column_degenerat___8 Other vertebral column degenerative disorders |
| | 30 | [metabolic_disorders]<br><br>Show the field ONLY if:<br>[aquired_abnormalitites] = '2' | Metabolic disorders | radio<br>1 Deficiencies (Vitamin B12, Folate, Copper, Vitamin D (Rickets))<br>2 Osteoporosis<br>3 Paget's disease<br>4 Osteomalacia<br>5 Other metabolic disorders |
| | 31 | [vascular_disorders]<br><br>Show the field ONLY if:<br>[aquired_abnormalitites] = '3' | Vascular disorders | radio<br>1 Haemorrhage (Epidural haematoma)<br>2 Vascular malformations (AV fistula, AVM)<br>3 Ischaemia |
| | 32 | [immunologic_diseases]<br><br>Show the field ONLY if:<br>[aquired_abnormalitites] = '4' | Inflammatory and auto-immune diseases | radio<br>1 Demyelination (MS, NMO, Transverse myelitis)<br>2 Collagen vascular disease (SLE, Sogren's, RA, AS, Vasculitis)<br>3 Sarcoidosis<br>4 Paraneoplastic<br>5 Arachnoiditis |

| | | | | |
|---|---|---|---|---|
| | | | | 6 Others |
| | 33 | [demyelination]<br><br>Show the field ONLY if:<br>[immunologic_diseases] = '1' | Demyelination | radio<br>1 Transverse Myelitis<br>2 Multiple sclerosis<br>3 Neuromyelitis Optica |
| | 34 | [neoplastic]<br><br>Show the field ONLY if:<br>[aquired_abnormalitites] = '7' | Neoplastic | radio<br>1 Benign<br>2 Malignant |
| | 35 | [benign]<br><br>Show the field ONLY if:<br>[neoplastic] = '1' | Benign | radio<br>1 Primary vertebral lesions (Osteoma, Osteochondroma, Osteoid osteoma, Haemangioma, Aneurysmal bone cyst)<br>2 Extradural space (Lipoma)<br>3 Intradural/Extramedullary (Neurofibroma, Meningioma, Schwannomas, Chordoma-benign)<br>4 Intramedullary (Astrocytoma-benign, Oligodendroglioma, Ependymoma, Cavernoma)<br>5 Other benign |
| | 36 | [malignant]<br><br>Show the field ONLY if:<br>[neoplastic] = '2' | Malignant | radio<br>1 Neural (Chordoma-malignant, Astrocytoma-malignant)<br>2 Primary vertebral lesions (Osteosarcoma, etc.)<br>3 Secondary vertebral lesions (Breast, Bronchus, Lung, Prostate, Renal, Thyroid, Ewing's sarcoma, Melanoma, etc.)<br>4 Haematological (Myeloma, Leukaemia, Non-Hodgkins Lymphoma, Hodgkin's lymphoma)<br>5 Other malignant |
| | 37 | [inflammation_or_infections] | Infections | radio |

| | | | | |
|---|---|---|---|---|
| | | Show the field ONLY if:<br>[aquired_abnormalitites] = '8' | | 1 Viral- Herpes group (Herpes simplex, Herpes zoster, CMV, Epstein-Barr)<br>2 Viral- Retrovirus (HIV, HTLV-1)<br>3 Viral- Enterovirus (Polio virus, Coxsackievirus, etc.)<br>4 Viral- others<br>5 Bacterial- Staph aureus [Extradural abscess]<br>6 Bacterial- Streptococcal [Extradural abscess]<br>7 Bacterial- Mycobacterium TB<br>8 Bacterial, Brucellosis<br>9 Bacterial- others<br>10 Spirochaetal- Treponema pallidum<br>11 Fungal (Cryptococcal, Actinomycosis, etc.)<br>12 Parasitic (Cysticercosis, Hydatid, Toxoplasmosis, Schistosomiasis, etc.) |
| | 38 | [timeframe_of_clinical_symp]<br>Show the field ONLY if:<br>[type_of_injury] = '2' | Timeframe of clinical symptoms onset | radio<br>1 Acute (less than 1 day)<br>2 Sub-acute (more than 1 day but less than 7 days)<br>3 Prolonged (more than 7 days but less than 1 month)<br>4 Lengthy (more than 1 month) |
| | 39 | [level_of_injury] | Level of Spinal Cord Injury | checkbox, Required<br>7 level_of_injury___7 Upper Cervical C1 - C4<br>8 level_of_injury___8 Lower Cervical C5 - C8<br>9 level_of_injury___9 Upper Thoracic T1 - T6<br>10 level_of_injury___10 Lower Thoracic T7 - T12<br>11 level_of_injury___11 Lumbar (Conus Medullaris) L1 - L2<br>12 level_of_injury___12 Cauda Equina Syndrome L3 - L5 |

| | | | | |
|---|---|---|---|---|
| | | | | 13 \| level_of_injury___13 \| Sacral S1 - S5 |
| | 40 | [level_of_sci] | Level of Spinal Cord Injury (detailed) | checkbox |

| | | |
|---|---|---|
| 63 | level_of_sci___63 | C1 |
| 64 | level_of_sci___64 | C2 |
| 65 | level_of_sci___65 | C3 |
| 66 | level_of_sci___66 | C4 |
| 67 | level_of_sci___67 | C5 |
| 68 | level_of_sci___68 | C6 |
| 69 | level_of_sci___69 | C7 |
| 70 | level_of_sci___70 | C8 |
| 71 | level_of_sci___71 | T1 |
| 72 | level_of_sci___72 | T2 |
| 73 | level_of_sci___73 | T3 |
| 74 | level_of_sci___74 | T4 |
| 75 | level_of_sci___75 | T5 |
| 76 | level_of_sci___76 | T6 |
| 77 | level_of_sci___77 | T7 |
| 78 | level_of_sci___78 | T8 |
| 79 | level_of_sci___79 | T9 |
| 80 | level_of_sci___80 | T10 |
| 81 | level_of_sci___81 | T11 |
| 82 | level_of_sci___82 | T12 |
| 83 | level_of_sci___83 | L1 |
| 84 | level_of_sci___84 | L2 |
| 85 | level_of_sci___85 | L3, L4, L5: Cauda Equina Syndrome |
| 86 | level_of_sci___86 | S1, S2, S3, S4, S5: Sacral sparring |

| | | | | |
|---|---|---|---|---|
| | 41 | [completeness] | Completeness of SCI | radio<br>1 Complete<br>2 Incomplete |
| | 42 | [type_of_spinal_cord_injury] | Type of spinal cord injury | radio<br>1 Paraplegic<br>2 Paraparesis<br>3 Tetraplegic<br>4 Tetraparesis<br>5 Hemiplegic<br>6 Hemiparesis |
| | 43 | [sci_syndromes] | SCI syndromes | radio<br>1 Anterior Cord Syndrome<br>2 Posterior Cord Syndrome<br>3 Central Cord Syndrome<br>4 Brown-Séquard Syndrome<br>5 Cauda equina syndrome |
| | 44 | [vertebral_fracture] | Vertebral fracture | radio<br>1 Yes<br>2 No<br><br>Custom alignment: RH |
| | 45 | [number_of_fractured_verteb]<br><br>Show the field ONLY if:<br>[vertebral_fracture] = '1' | Number of fractured vertebra(e) | text (number, Min: 1, Max: 34) |
| | 46 | [fractured_vertebrae]<br><br>Show the field ONLY if:<br>[vertebral_fracture] = '1' | Fractured vertebra(e) | checkbox<br>31 fractured_vertebrae___31 C1<br>32 fractured_vertebrae___32 C2 |

| | | |
|---|---|---|
| 33 | fractured_vertebrae___33 | C3 |
| 34 | fractured_vertebrae___34 | C4 |
| 35 | fractured_vertebrae___35 | C5 |
| 36 | fractured_vertebrae___36 | C6 |
| 37 | fractured_vertebrae___37 | C7 |
| 38 | fractured_vertebrae___38 | T1 |
| 39 | fractured_vertebrae___39 | T2 |
| 40 | fractured_vertebrae___40 | T3 |
| 41 | fractured_vertebrae___41 | T4 |
| 42 | fractured_vertebrae___42 | T5 |
| 43 | fractured_vertebrae___43 | T6 |
| 44 | fractured_vertebrae___44 | T7 |
| 45 | fractured_vertebrae___45 | T8 |
| 46 | fractured_vertebrae___46 | T9 |
| 47 | fractured_vertebrae___47 | T10 |
| 48 | fractured_vertebrae___48 | T11 |
| 49 | fractured_vertebrae___49 | T12 |
| 50 | fractured_vertebrae___50 | L1 |
| 51 | fractured_vertebrae___51 | L2 |
| 52 | fractured_vertebrae___52 | L3 |
| 53 | fractured_vertebrae___53 | L4 |
| 54 | fractured_vertebrae___54 | L5 |
| 55 | fractured_vertebrae___55 | S1 |
| 56 | fractured_vertebrae___56 | S2 |
| 57 | fractured_vertebrae___57 | S3 |
| 58 | fractured_vertebrae___58 | S4 |
| 59 | fractured_vertebrae___59 | S5 |

| | | | | |
|---|---|---|---|---|
| | | | | 60 \| fractured_vertebrae___60 \| Co1 |
| | 47 | [icd_code] | ICD code | text |
| | 48 | [pre_hospital_data]<br><br>Show the field ONLY if:<br>[type_of_injury] = '1' | Pre-hospital data | checkbox<br>1 \| pre_hospital_data___1 \| Cardiac arrest during transport<br>2 \| pre_hospital_data___2 \| CPR during transport<br>3 \| pre_hospital_data___3 \| Orotracheal intubation<br>4 \| pre_hospital_data___4 \| Unknown |
| | 49 | [pre_hospital_immobilizatio]<br><br>Show the field ONLY if:<br>[type_of_injury] = '1' | Pre-hospital immobilization | checkbox<br>1 \| pre_hospital_immobilizatio___1 \| Cervical fixation<br>2 \| pre_hospital_immobilizatio___2 \| Spinal fixation<br>3 \| pre_hospital_immobilizatio___3 \| Limb fixation<br>4 \| pre_hospital_immobilizatio___4 \| None |
| | 50 | [associated_injury]<br><br>Show the field ONLY if:<br>[type_of_injury] = '1' | Associated injuries | checkbox<br>1 \| associated_injury___1 \| Traumatic brain injury<br>2 \| associated_injury___2 \| Burn<br>3 \| associated_injury___3 \| Extremity fracture<br>4 \| associated_injury___4 \| Internal organ damage |
| | 51 | [hospital_of_admission] | Hospital of admission | text |
| | 52 | [admission_to_hospital_time] | Date of first admission | text (date_dmy) |
| | 53 | [time_of_first_admission_to]<br><br>Show the field ONLY if:<br>[timeframe_of_clinical_symp] = '1' or [type_of_injury] = '1' | Time of first admission | text (time) |
| | 54 | [transport_to_the_hospital] | Transport to the hospital | radio<br>1 \| Emergency Medical Service (EMS)<br>2 \| Helicopter |

| | | | | |
|---|---|---|---|---|
| | | | | 3 Personal vehicle<br>4 Other |
| | 55 | [asia_score_at_the_time_of] | ASIA Score (at the time of admission) | radio<br>1 A<br>2 B<br>3 C<br>4 D<br>5 E<br><br>Custom alignment: RH |
| | 56 | [gcs_at_the_time_of_admissi]<br>Show the field ONLY if:<br>[type_of_injury] = '1' | GCS (at the time of admission) | text (number, Min: 3, Max: 15) |
| | 57 | [department_ward_of_admissi] | Department of admission | radio<br>1 Neurological surgery<br>2 Orthopeadic surgery<br>3 Neurology<br>4 Others<br>5 Patient deceased in ED |
| | 58 | [medical_complication_durin] | Medical complications at the time of admission | checkbox<br>1 medical_complication_durin___1 CSF leakage<br>2 medical_complication_durin___2 Pressure ulcer<br>3 medical_complication_durin___3 Fever<br>4 medical_complication_durin___4 Mechanical ventilation<br>5 medical_complication_durin___5 Spasticity |
| | 59 | [medical_history] | Medical History | checkbox<br>1 medical_history___1 Cardiovascular diseases |

| | | | | |
|---|---|---|---|---|
| | | | | 2 \| medical_history___2 \| Pulmonary disease<br>3 \| medical_history___3 \| Other medical conditions |
| | 60 | [cardiovascular_diseases_hi]<br><br>Show the field ONLY if:<br>[medical_history(1)] = '1' | Cardiovascular diseases history | checkbox<br>1 \| cardiovascular_diseases_hi___1 \| CVA (Cerebrovascular accident)<br>2 \| cardiovascular_diseases_hi___2 \| MI (Miocardial Infarction)<br>3 \| cardiovascular_diseases_hi___3 \| Peripheral vascular disease<br>4 \| cardiovascular_diseases_hi___4 \| Hypertension<br>5 \| cardiovascular_diseases_hi___5 \| Hypotension<br>6 \| cardiovascular_diseases_hi___6 \| CHF (Chronic Heart Failiure)<br>7 \| cardiovascular_diseases_hi___7 \| CAD (Coronary artery disease)<br>8 \| cardiovascular_diseases_hi___8 \| DVT (Deep vein thrombosis)<br>9 \| cardiovascular_diseases_hi___9 \| Orthostatic hypotension<br>10 \| cardiovascular_diseases_hi___10 \| Arrhythmias<br>11 \| cardiovascular_diseases_hi___11 \| Cardiac Pacemaker<br>12 \| cardiovascular_diseases_hi___12 \| Cardiac surgery<br>13 \| cardiovascular_diseases_hi___13 \| Hypercholesterolemia<br>14 \| cardiovascular_diseases_hi___14 \| Other cardiac disorders |
| | 61 | [pulmonary_diseases_history]<br><br>Show the field ONLY if:<br>[medical_history(2)] = '1' | Pulmonary diseases history | checkbox<br>1 \| pulmonary_diseases_history___1 \| Asthma<br>2 \| pulmonary_diseases_history___2 \| COPD<br>3 \| pulmonary_diseases_history___3 \| Sleep apnea |

| | | | | |
|---|---|---|---|---|
| | | | | 4 \| pulmonary_diseases_history___4 \| Smoking history<br>5 \| pulmonary_diseases_history___5 \| Unknown |
| | 62 | [other_medical_conditions]<br><br>Show the field ONLY if:<br>[medical_history(3)] = '1' | Other medical conditions | checkbox<br>1 \| other_medical_conditions___1 \| Diabetes Mellitus<br>2 \| other_medical_conditions___2 \| ESRD<br>3 \| other_medical_conditions___3 \| Chronic kidney disease<br>4 \| other_medical_conditions___4 \| Cirrhosis<br>5 \| other_medical_conditions___5 \| Dementia<br>6 \| other_medical_conditions___6 \| AIDS<br>7 \| other_medical_conditions___7 \| Rheumatologic diseases<br>8 \| other_medical_conditions___8 \| Epilepsy<br>9 \| other_medical_conditions___9 \| Depression<br>10 \| other_medical_conditions___10 \| Malignancy<br>11 \| other_medical_conditions___11 \| Thyroid dysfunction<br>12 \| other_medical_conditions___12 \| Others |
| | 63 | [surgical_intervention_for_sci] | Surgical intervention for SCI | yesno<br>1 \| Yes<br>0 \| No<br><br>Custom alignment: RH |
| | 64 | [surgical_intervention]<br><br>Show the field ONLY if:<br>[surgical_intervention_for_sci] = '1' | Type of surgical intervention | checkbox<br>1 \| surgical_intervention___1 \| Spinal fixation and fusion<br>2 \| surgical_intervention___2 \| Others<br><br>Custom alignment: RH |
| | 65 | [spinal_fixation_and_fusion] | Spinal fixation and fusion | radio |

| | | | | |
|---|---|---|---|---|
| | | Show the field ONLY if:<br>[surgical_intervention(1)] = '1' | | 1 One level<br>2 Two levels<br>3 ≧Three levels<br>4 Undetermined |
| | 66 | [other_surgical_interventions]<br>Show the field ONLY if:<br>[surgical_intervention(2)] = '1' | Please specify: | text<br>Custom alignment: RH |
| | 67 | [standard_dose_prednisolone] | Did the patient receive corticosteroid at the time of admission? | yesno<br>1 Yes<br>0 No<br>Custom alignment: RH |
| | 68 | [non_surgical_intervention] | Non-surgical interventions | checkbox<br>1 non_surgical_intervention___1 External immobilizing device<br>2 non_surgical_intervention___2 Enforced bed-rest |
| | 69 | [incontinence] | Incontinence | checkbox<br>1 incontinence___1 Bladder incontinence/retention<br>2 incontinence___2 Bowel incontinence |
| | 70 | [date_of_discharge]<br>Show the field ONLY if:<br>[type_of_injury] = '1' | Date of discharge of the first admission | text (date_dmy) |
| | 71 | [patient_condition_at_the_t] | Condition at the time of discharge | radio<br>1 Good Recovery (Resumption of normal life with the capacity to work even if pre-injury status has not been achieved. Some patients have minor neurological or psychological deficits)<br>2 Moderate Disability (Patients have some disability such as aphasia, hemiparesis or epilepsy and/or |

| | | | | |
|---|---|---|---|---|
| | | | | deficits of memory or personality but are able to look after themselves. They are independent at home but dependent outside)<br>3 Severe Disability (Patients are dependent to daily support for mental and/or physical disability)<br>4 Unresponsive Wakefulness or Vegetative State (Condition of unawareness with only reflexive responses but with periods of spontaneous eye opening)<br>5 Deceased |
| | 72 | [icu_length_of_stay_days] | ICU length of stay (days) | text (number, Min: 0, Max: 1000) |
| | 73 | [survival_status] | Latest available survival status | radio<br>1 Alive<br>2 Dead<br>3 Lost to follow up (unknown) |
| | 74 | [auxiliary_medical_equipmen] | Auxiliary medical equipment | checkbox<br>1 auxiliary_medical_equipmen___1 Cervical external immobilization<br>2 auxiliary_medical_equipmen___2 Thoracolumbar or sacral external immobilization<br>3 auxiliary_medical_equipmen___3 Tracheostomy<br>4 auxiliary_medical_equipmen___4 Mechanical ventilation |
| | 75 | [death_date]<br>Show the field ONLY if:<br>[survival_status] = '2' | Death date | text (date_dmy) |
| | 76 | [causes_of_death]<br>Show the field ONLY if:<br>[survival_status] = '2' | Cause of death | text |

| | | | | |
|---|---|---|---|---|
| | 77 | [data_extractor] | Data Extractor | text |
| | 78 | [my_first_instrument_complete] | Section Header: *Form Status*<br>Complete? | dropdown<br>0 Incomplete<br>1 Unverified<br>2 Complete |

Adding new Record number **1**.

| | |
|---|---|
| **Record number** | 1 |
| **First Name** * must provide value | Paper |
| **Last Name** * must provide value | Paper |
| **Patient ID** | Paper |
| **Sex** * must provide value | ○ Male<br>◉ Female<br>○ Others<br>reset |
| **Birth date** | 29-04-2025 Today D-M-Y |
| **Nationality** | Austrian |
| **Marital status (at the time of injury)** | ◉ Single<br>○ Married<br>○ Widow(er)<br>○ Divorced<br>○ Unknown<br>reset |
| **Education level** | ○ None (illiterate)<br>○ Compulsory schooling (9 years)<br>◉ High school (9-12 years)<br>○ College degree (Bachelor and Master)<br>○ Graduate degree (Doctorate) and higher<br>○ Unknown<br>reset |
| **Telephone number (Including country code)** * must provide value | Rock |
| **Address** | Rock |
| **City of residence at the time of injury** | Scissors |
| **City where the injury happened** * must provide value | Rock |
| **Postal code** | Rock |
| **Type of Injury** * must provide value | ◉ Traumatic<br>○ Non-traumatic<br>reset |
| **Traumatic cause** | ○ Transport injuries<br>○ Falls<br>○ Sports<br>○ Drowning<br>○ Fire and hot substances<br>○ Conflict, assault or violence<br>○ Gunshot wounds<br>○ Suicide<br>○ Disasters<br>○ Occupational<br>◉ Others<br>reset |
| **Injury date** | 29-04-2025 Today D-M-Y |
| **Injury time** | 12:34 Now H:M |
| **Day of injury** | ○ Monday<br>○ Tuesday<br>○ Wednesday<br>○ Thursday<br>◉ Friday<br>○ Saturday<br>○ Sunday<br>reset |

**Activity when the injury happened**

- ( ) Sports activity
- ( ) Leisure activity
- (•) Working for income activity
- ( ) Other activities (resting, sleeping, eating, or engaging)
- ( ) Unspecified activities

reset

**Place where injury happened**

- ( ) Home
- ( ) Residential institution
- ( ) Sports and athletics area
- ( ) School, other institution and public administrative area
- ( ) Street and highway
- ( ) Trade and service area
- ( ) Industrial and construction area
- ( ) Farming land
- (•) Other specified places
- ( ) Unspecified place

reset

**Mechanism of injury**

- ( ) Penetrating
- (•) Blunt
- ( ) Undetermined

reset

**Safety devices at the time of injury**

- (•) Helmet
- ( ) Seat belt
- ( ) Airbag
- ( ) Protective clothing
- ( ) Child seat
- ( ) None
- ( ) Unknown

reset

**Level of Spinal Cord Injury**

* must provide value

- [ ] Upper Cervical C1 - C4
- [x] Lower Cervical C5 - C8
- [ ] Upper Thoracic T1 - T6
- [ ] Lower Thoracic T7 - T12
- [ ] Lumbar (Conus Medullaris) L1 - L2
- [ ] Cauda Equina Syndrome L3 - L5
- [ ] Sacral S1 - S5

**Level of Spinal Cord Injury (detailed)**

- [ ] C1
- [ ] C2
- [ ] C3
- [ ] C4
- [ ] C5
- [x] C6
- [x] C7
- [ ] C8
- [ ] T1
- [ ] T2
- [ ] T3
- [ ] T4
- [ ] T5
- [ ] T6
- [ ] T7
- [ ] T8
- [ ] T9
- [ ] T10
- [ ] T11
- [ ] T12
- [ ] L1
- [ ] L2
- [ ] L3, L4, L5: Cauda Equina Syndrome
- [ ] S1, S2, S3, S4, S5: Sacral sparring

**Completeness of SCI**

- (•) Complete
- ( ) Incomplete

reset

**Type of spinal cord injury**

- (•) Paraplegic
- ( ) Paraparesis
- ( ) Tetraplegic
- ( ) Tetraparesis
- ( ) Hemiplegic
- ( ) Hemiparesis

reset

| | | |
|---|---|---|
| **SCI syndromes** | ◯ Anterior Cord Syndrome<br>◉ Posterior Cord Syndrome<br>◯ Central Cord Syndrome<br>◯ Brown-Séquard Syndrome<br>◯ Cauda equina syndrome | reset |
| **Vertebral fracture** | ◉ Yes ◯ No | reset |
| **Number of fractured vertebra(e)** | 1 | |
| **Fractured vertebra(e)** | ☐ C1<br>☐ C2<br>☐ C3<br>☐ C4<br>☐ C5<br>☑ C6<br>☐ C7<br>☐ T1<br>☐ T2<br>☐ T3<br>☐ T4<br>☐ T5<br>☐ T6<br>☐ T7<br>☐ T8<br>☐ T9<br>☐ T10<br>☐ T11<br>☐ T12<br>☐ L1<br>☐ L2<br>☐ L3<br>☐ L4<br>☐ L5<br>☐ S1<br>☐ S2<br>☐ S3<br>☐ S4<br>☐ S5<br>☐ Co1 | |
| **ICD code** | Paper | |
| **Pre-hospital data** | ☑ Cardiac arrest during transport<br>☐ CPR during transport<br>☐ Orotracheal intubation<br>☐ Unknown | |
| **Pre-hospital immobilization** | ☐ Cervical fixation<br>☐ Spinal fixation<br>☑ Limb fixation<br>☐ None | |
| **Associated injuries** | ☐ Traumatic brain injury<br>☐ Burn<br>☐ Extremity fracture<br>☑ Internal organ damage | |
| **Hospital of admission** | Scissors | |
| **Date of first admission** | 29-04-2025 Today D-M-Y | |
| **Time of first admission** | 12:34 Now H:M | |
| **Transport to the hospital** | ◉ Emergency Medical Service (EMS)<br>◯ Helicopter<br>◯ Personal vehicle<br>◯ Other | reset |
| **ASIA Score (at the time of admission)** | ◯ A ◉ B ◯ C ◯ D ◯ E | reset |

| | |
|---|---|
| **GCS (at the time of admission)** | 3 |
| **Department of admission** | ◉ Neurological surgery<br>○ Orthopeadic surgery<br>○ Neurology<br>○ Others<br>○ Patient deceased in ED<br>reset |
| **Medical complications at the time of admission** | ☑ CSF leakage<br>☐ Pressure ulcer<br>☐ Fever<br>☐ Mechanical ventilation<br>☐ Spasticity |
| **Medical History** | ☐ Cardiovascular diseases<br>☑ Pulmonary disease<br>☐ Other medical conditions |
| **Pulmonary diseases history** | ☑ Asthma<br>☐ COPD<br>☐ Sleep apnea<br>☐ Smoking history<br>☐ Unknown |
| **Surgical intervention for SCI** | ◉ Yes ○ No<br>reset |
| **Type of surgical intervention** | ☐ Spinal fixation and fusion ☑ Others |
| **Please specify:** | Scissors |
| **Did the patient receive corticosteroid at the time of admission?** | ○ Yes ◉ No<br>reset |
| **Non-surgical interventions** | ☐ External immobilizing device<br>☑ Enforced bed-rest |
| **Incontinence** | ☑ Bladder incontinence/retention<br>☐ Bowel incontinence |
| **Date of discharge of the first admission** | 29-04-2025 Today D-M-Y |
| **Condition at the time of discharge** | ○ Good Recovery (Resumption of normal life with the capacity to work even if pre-injury status has not been achieved. Some patients have minor neurological or psychological deficits)<br>○ Moderate Disability (Patients have some disability such as aphasia, hemiparesis or epilepsy and/or deficits of memory or personality but are able to look after themselves. They are independent at home but dependent outside)<br>◉ Severe Disability (Patients are dependent to daily support for mental and/or physical disability)<br>○ Unresponsive Wakefulness or Vegetative State (Condition of unawareness with only reflexive responses but with periods of spontaneous eye opening)<br>○ Deceased<br>reset |
| **ICU length of stay (days)** | 5 |
| **Latest available survival status** | ◉ Alive<br>○ Dead<br>○ Lost to follow up (unknown)<br>reset |
| **Auxiliary medical equipment** | ☐ Cervical external immobilization<br>☑ Thoracolumbar or sacral external immobilization<br>☐ Tracheostomy<br>☐ Mechanical ventilation |
| **Data Extractor** | Scissors |
| **Form Status** | |

**Complete?**

Complete

**Lock this instrument?**

If locked, no user will be able to modify this instrument for this record until someone with Instrument Level Lock/Unlock privileges unlocks it.

Lock

Save & Exit Form

Save & ...

– Cancel –
